# *Pneumocystis Jirovecii* Pneumonia Diagnosis *via* Metagenomic Next-Generation Sequencing

**DOI:** 10.3389/fmed.2022.812005

**Published:** 2022-03-09

**Authors:** Xiaoxiao Lu, Jianhui Zhang, Wentao Ma, Lihua Xing, Hanbing Ning, Mengying Yao

**Affiliations:** ^1^Department of Respiratory and Critical Care Medicine, The First Affiliated Hospital of Zhengzhou University, Zhengzhou, China; ^2^Gene Hospital of Henan Province, Precision Medicine Center, The First Affiliated Hospital of Zhengzhou University, Zhengzhou, China; ^3^Department of Digestive Diseases, The First Affiliated Hospital of Zhengzhou University, Zhengzhou, China

**Keywords:** *Pneumocystis jirovecii* pneumonia, *P. jirovecii*, GMS, mNGS, diagnosis

## Abstract

The incidence of non-HIV-infected Pneumocystis Jirovecii Pneumonia (PJP) is increasing. The prognosis for non-HIV PJP is poor and diagnostic tests are of lower sensitivity in non-HIV patients. Metagenomic next-generation sequencing (mNGS) was compared with routine detection assays, including Gomori methenamine silver (GMS) stain and polymerase chain reaction (PCR) technique. Specimens of 4 bronchoalveolar lavages (BAL) and 1 lung tissue samples were obtained from 4 non-HIV patients from our hospitals. Although both GMS and mNGS were positive for *P. jirovecii* with PCR as positive control, the testing time of mNGS was obviously shorter than GMS. Compared with the traditional GMS method, mNGS has absolute advantages. However, the issue with PJP presentations having atypical symptoms and ambiguous imaging features persists. Hence, the disease can easily be ignored. Secondly, PJP progresses rapidly in non-HIV-infected patients and can cause severe respiratory failure with unfavorable prognosis. This study affirms that mNGS can be used to quickly and accurately diagnose PJP, but a combination of clinical judgement of symptoms, laboratory testing, and imaging examination is required to make a comprehensive judgment along with mNGS test results.

## Introduction

Pneumocystis carinii pneumonia (PJP) is a potentially life-threatening opportunistic pathogenic fungal infection caused by *P. jirovecii*. Initially, PJP was the most common opportunistic infection in people with HIV infection or Acquired Immune Deficiency Syndrome (AIDS). However, in recent years, the incidence of PJP in patients with HIV has decreased due to the use of highly active antiretroviral therapy (HAART) and preventive treatment against *P. jirovecii*. PJP was known to be a rare non-HIV illness that occur in immunocompromised individuals even before advent of HIV. It had reported mortality rates that ranged from 19.6 to 52% in non-HIV patients (1–3), which is significantly higher than in HIV-infected patients (3–5). In contrast to HIV-infected patients, there is evidence for a more acute onset of symptoms, faster progression of disease, poorer outcome, higher mortality, and higher risk of coinfections (6–10).

*P. jirovecii* is an atypical fungus. It does not grow on culture media *in vitro*. Currently, the gold standard for the diagnosis of PJP is the discovery of characteristic cysts or trophoblasts by microscopic staining examination of lower respiratory tract specimens. Although the traditional microscopic examination is simple and inexpensive, the sensitivity of microscopy is lower in non-HIV-infected compared to HIV-infected patients due to a lower fungal load (11, 12). In recent years, real-time PCR has been recommended for the routine diagnosis of PJP (A-II:) by the Fifth European Conference on Infections in Leukemia (ECIL-5) (12).

With the development of molecular biology, the value of metagenome next-generation sequencing (mNGS), which can be used for the co-pathogens in mixed pulmonary infection, has been recognized. The utility of mNGS in etiological detection and identification of respiratory tract infections has recently been demonstrated (13), especially as it can identify the pathogens that cannot be cultured *in vitro*, such as *pneumocystis*. In this study, we identified PJP with the technology of mNGS, proving its important role in PJP diagnosis.

## Materials and Methods

The study began on August 17, 2020. We performed traditional microbiological testing and mNGS on samples from patients with fever of unknown origin and suspected *P. jirovecii* infection. Furthermore, we collected patients' baseline information, clinical features, laboratory and imaging examination results, diagnoses, treatments, and outcomes. In the end, we confirmed four cases of PJP using PCR, Gomori methenamine silver (GMS), and mNGS.

### PCR Assay

Total RNA was isolated from cells using Trizol as reagent (Invitrogen, Carlsbad, CA, USA) according to the manufacturer's protocol. Total RNA (1 μg) was reverse transcribed using a Reverse Transcription kit according to the manufacturer's protocol (Promega, WI, USA). cDNA (2 μl) was then amplified using the following conditions: PTEN: 40 cycles of denaturation for 15 s at 95°C; annealing for 15 s at 55°C; and extension for 30 s at 72°C. The PCR product for each sample was separated by 2% agarose gel electrophoresis and visualized by ultraviolet (UV) light. The sequence of the primer pairs used is listed as follows: PjF1 (5′-GCATTTTTCAAACATCTGTG-3′) and PjR1 (5′-CGCGAGAGCCMAGAGATCC-3′). The *P. jirovecii* plasmid mRNA expression level was employed as positive control.

### GMS Stain Assay

The bronchoalveolar lavage (BAL) sample was directly centrifuged. The pellet was smeared, dried, and fixed with methanol. The slides were stained with GMS and examined by different experienced microscopists not apprised of the identity of the sample. PJP cysts are shown black or dark brown, round, or quasi-round.

### mNGS Protocol

The microbial community DNA was extracted using a custom kit. After qualification by quality control (QC), the DNA was randomly fragmented and made into a sequencing library. The qualified libraries were sequenced on NextSeq 550Dx platform (illumine, USA) with a pair-end sequencing length of 75 bp. Bioinformatics analysis was performed on samples with raw data >10 million. By removing low-quality and low complexity regions, the human host sequence was mapped to the human reference genome (hg19) using Burrows-Wheeler alignment to remove human reads. The high-quality data were classified by simultaneously aligned to four Microbial Genome Databases, including viruses, bacteria, fungi, and parasites. The databases were downloaded from NCBI (https://www.ncbi.nlm.nih.gov/genomes/). A microbe was considered clinically significant when its relative abundance at the species level was >30% and its pulmonary pathogenicity has been proven in literature.

## Results

**Figure 1:**
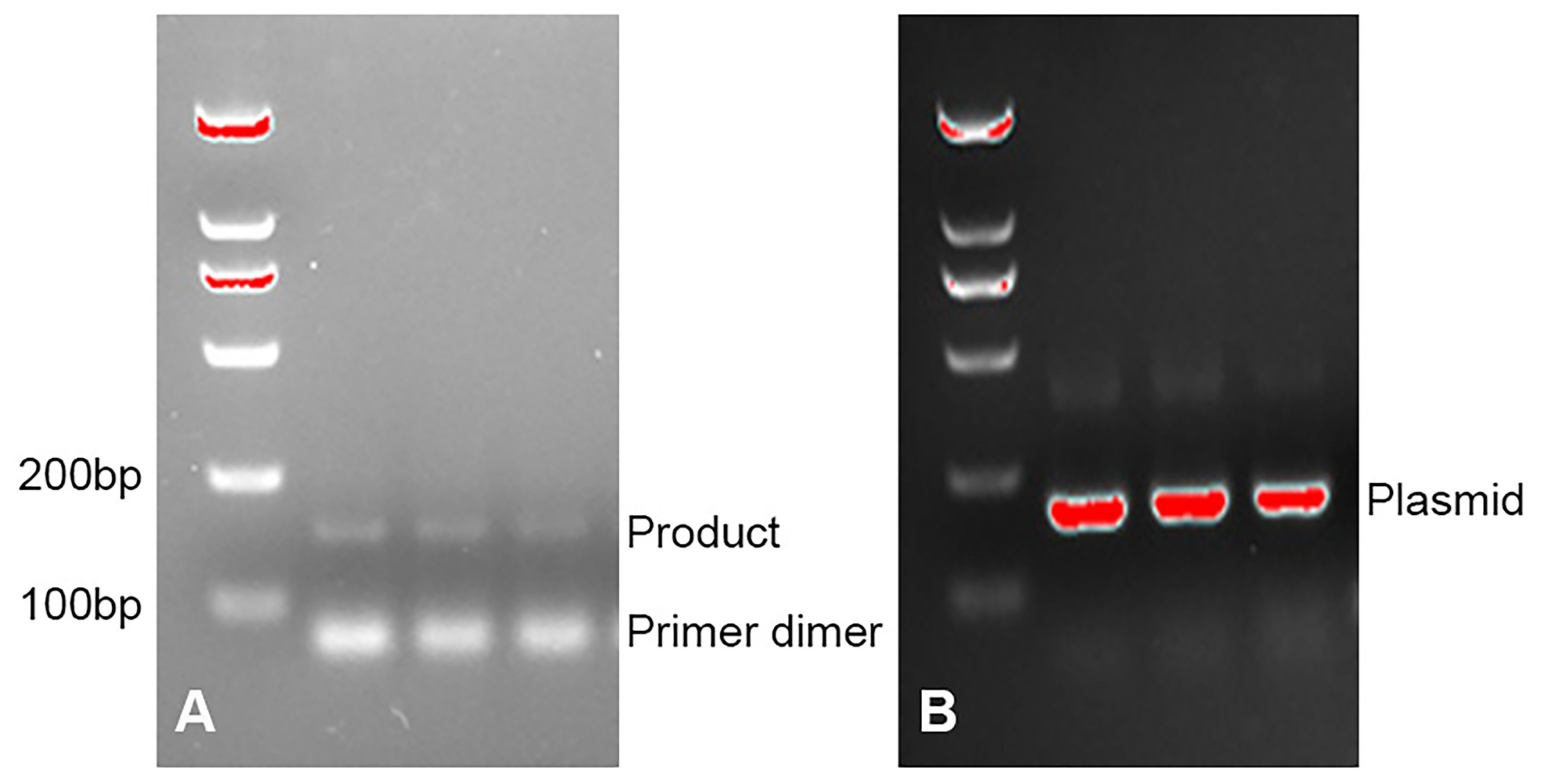
**(A)** PCR detection for *P. jirovecii* DNA on bronchoalveolar lavage fluid (BALF) specimens. **(B)** PCR detection for plasmid.

In this study, patients who were suspected to have PJP by two or more expert professors were assessed by PCR, GMS, and mNGS analysis. We discovered a total of four patients with PJP. These patients had common clinical symptoms of chest tightness, and dyspnea and all had a previous condition that required long-term glucocorticoid or immunosuppressant (ISA) use (Tables 1, 2). Bronchoalveolar lavage fluid (BALF) samples of three patients were examined by PCR as a positive control. As shown in Figure 1, PCR gave the positive yield for *P. jirovecii* detection. The patients' detailed information was described as follows.

**Table 1:** Clinical characteristics of patients.

**Patients NO./Sex/Age, year**	**Final diagnosis**	**Clinical presentation**	**Underlying disease**	**GC**	**ISA**
NO.1/female/78	PCP	fever, cough, sputum, chest tightness, dyspnea	MPA	Yes	Yes
NO.2/female/77	PCP	palpitations, dyspnea, chest tightness	AIH	Yes	Yes
NO.3/male/54	PCP	fever, chest tightness, dyspnea	NS	Yes	No
NO.4/male/58	PCP	fever, chest tightness, dyspnea	NS	Yes	Yes

*MPA, microscopic polyangiitis; AIH, autoimmune hepatitis; GC, glucocorticoids; ISA, immunosuppressant; NS, Nephrotic Syndrome*.

**Table 2:** Summary of laboratory test results and metagenomic next-generation sequencing (mNGS) results.

**Patient no**.	**Sample**	**Disease duration of time of diagnostic, day**	**Traditional microbiological testing results**	**mNGS testing results**	**mNGS sequence number**
1	BALF	33	*P. jirovecii*	*Nocardia neocaledoniensis, CMV, EBV, P. jirovecii*, *Aspergillus niger, Aspergillus welwitschiae*	20,564
2	BALF, lung tissue	15	*P. jirovecii Candida albicans, Pseudomonas aeruginosa*	*Klebsiella pneumoniae*	1,319 (BALF), 297 (lung tissue)
3	BALF	6	*Klebsiella pneumoniae*	*P. jirovecii Klebsiella pneumoniae*	34
4	BALF	14	*Candida glabrata*	*P. jirovecii*	279

*CMV, cytomegalovirus; EBV, Epstein Barr virus*.

### Case Descriptions

**Patient 1** was a 78-year-old female retiree from Henan Province, China who was admitted to Respiratory Intensive Care Unit (RICU) for “intermittent fever for 1 month and dyspnea for 10 days.” The main clinical symptoms of the patient were intermittent fever with a highest body temperature of 38.7°C, cough, sputum, chest tightness, dyspnea, dizzy, fatigue, and oral ulcer. She had been diagnosed with microscopic polyangiitis (MPA) for 3 years and had been using prednisone and azathioprine for a long time. She had hypertension, coronary artery disease (CAD), and previous cerebral infarction.

The following abnormal laboratory test results were obtained: white blood cell count, 2.5 × 10^9^ cells/L; red blood cell count, 2.38 × 10^12^ cells/L; hemoglobin, 85 g/L; lactate dehydrogenase, 429 U/L; procalcitonin, 0.117 ng/ml; C-reactive protein, 7.27 mg/L; erythrocyte sedimentation rate, 34 mm/h; Interleukin 6, 35.7 pg/ml; Interleukin 10, 44.57 pg/ml; KL-6, 933 U/ml; Coxsackie virus, EB virus, measles virus, and cytomegalovirus IgG positive; EB virus and cytomegalovirus IgM positive; percentage of total T lymphocytes, 90.71%; Absolute number of helper/inducible T lymphocytes, 33/ul; chest radiograph, multiple large flaps of exudate in both lungs.

**Figure 2:**
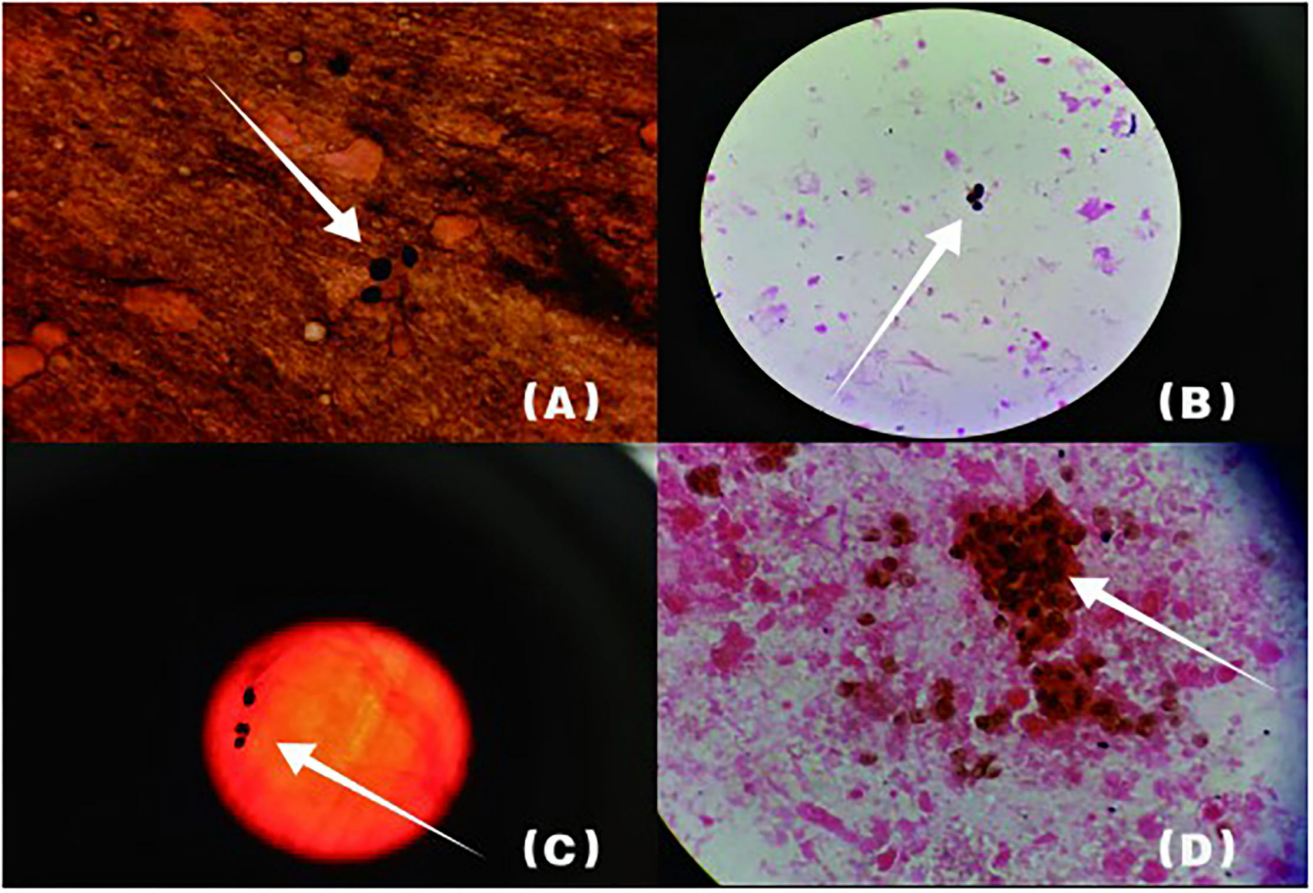
Arrowheads show *P. jirovecii* cysts which are round or oral. The cyst wall is darkened or brownish-black by GMS, while the content is almost colorless. **(B)** shows lung tissue specimen, **(A,C,D)** are BALF specimens.

BALF was collected for examination on the second day of admission. GMS (Figure 2) and mNGS all detected *P. jirovecii*, but the detection period of mNGS was shorter than that of GMS at 4 days. mNGS results showed that the sequence number of *P. jirovecii* was 20,564 (Table 2). Based on the GMS and mNGS results, the patient was diagnosed with PJP. However, the patient was admitted to RICU in serious condition and was given biapenem, voriconazole, gamma globulin, and methylprednisolone therapy in the early stage. Unfortunately, the patient's condition did not improve. Clinicians made a comprehensive judgment according to the results of mNGS and routine tests when we subsequently confirmed that the patient was infected with *P. jirovecii, Epstein Barr virus* (EBV), and *cytomegalovirus* (CMV). Compound sulfamethoxazole (SMZ-TMP) was empirically administered on the second day of admission. Even if the diagnosis was confirmed and targeted treatment was given, the patient's condition rapidly deteriorated and she developed severe respiratory failure and septic shock. There was still no improvement upon mechanical ventilation and other rescue treatments. Finally, the patient gave up on treatment and was discharged automatically on the fifth day of admission.

**Patient 2** was a 77-year-old female retiree from Henan Province, China who was admitted to the gastrointestinal department for “intermittent palpitations, chest distress for 10 days.” The main clinical symptoms of the patient were intermittent palpitations, chest tightness, and dyspnea. She had been diagnosed with autoimmune hepatitis (AIH) for 2 months and had been using prednisone and azathioprine for a long time. Her case was additionally complicated by hypertension, CHD, and a history of cerebral infarction. On the second day of admission, the patient's symptom of chest tightness worsened, and oxygen saturation was measured at 55–60%. The patient was urgently transferred to the RICU.

**Figure 3:**
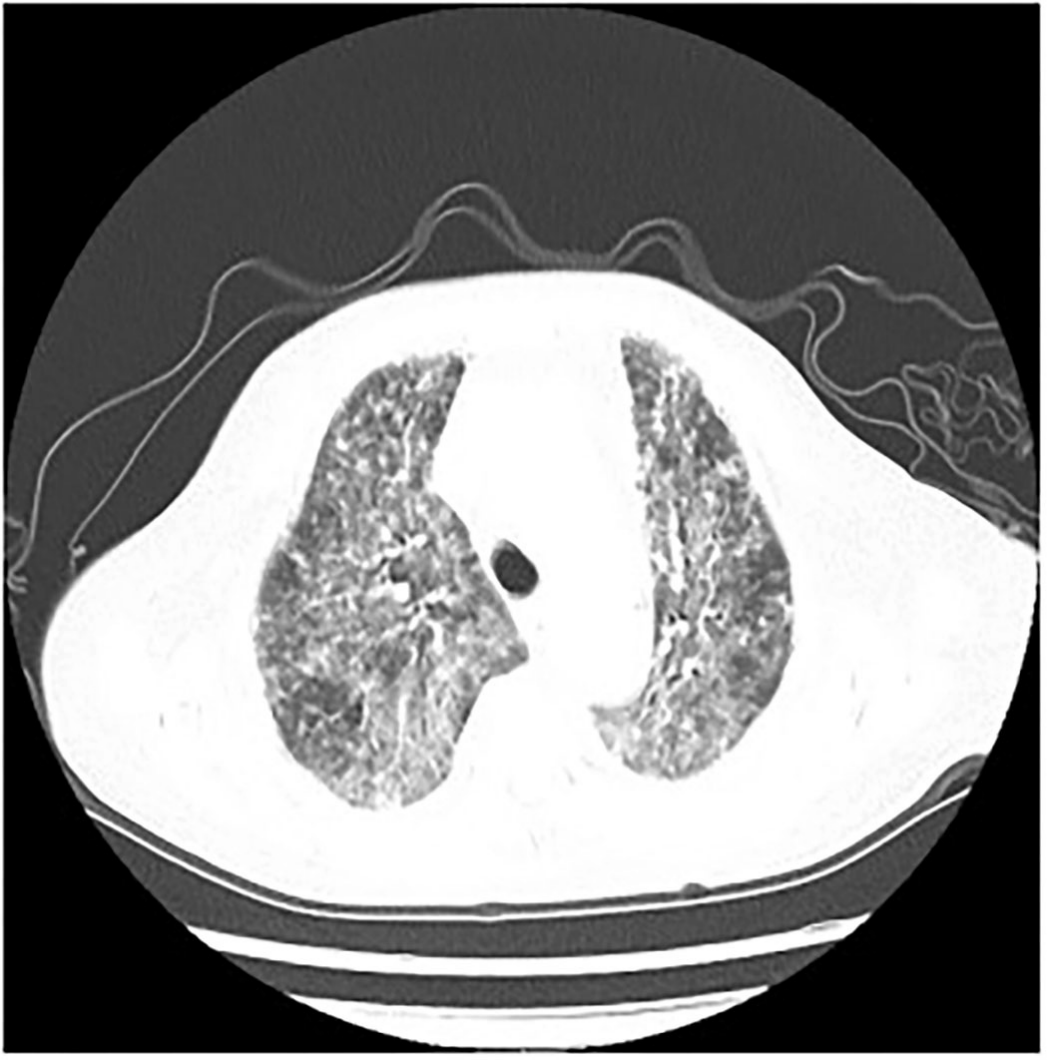
*P. jirovecii* appearances on Chest CT scan in patient 2 as multiple patchy and flocculent high-density shadows in both lungs.

The following abnormal laboratory test results were obtained: red blood cell count, 3.14 × 10^12^ cells/L; hemoglobin, 109 g/L; lactate dehydrogenase, 1,723 U/L; plasma β-(1, 3)-D glucan, 600 pg/ml; procalcitonin, 2.71 ng/ml; C-reactive protein, 106.94 mg/L; erythrocyte sedimentation rate, 21 mm/h; Interleukin 6, 17.8 pg/ml; Interleukin 10, 7.57 pg/ml; KL-6, 2,153 U/ml; Coxsackie virus, EB virus, measles virus, and cytomegalovirus IgG positive; percentage of total T lymphocytes, 48.77%; Absolute number of helper/inducible T lymphocytes, 32/μl; *Candida albicans* and *Pseudomonas aeruginosa* were detected by traditional microbiological testing. Chest CT showed multiple patchy and flocculent high-density shadows in both lungs (Figure 3). BALF was collected for examination on the second day of admission, and *P. jirovecii* was detected only upon mNGS. No *P. jirovecii* was found upon GMS. The results of mNGS detection in BALF showed that sequence number of *P. jirovecii* was 1,319 (Table 2). Subsequently, on the fourth day of admission, we collected lung tissue through fiberoptic bronchoscope for the same test. GMS (Figure 2B) and mNGS all detected *P. jirovecii*, but the detection period of mNGS was 1 day shorter than that of GMS. mNGS in lung tissue smear showed that sequence number of *P. jirovecii* was 297 (Table 2). Based on the GMS and mNGS results, the patient was diagnosed with PJP. SMZ-TMP had been empirically applied on the second day of admission. Originally, we gave patients biapenem, voriconazole, gamma globulin, and other symptomatic treatments. However, the progress of this patient was the same as that of the **patient 1**. Even after we gave mechanical ventilation, extracorporeal membrane oxygenation (ECMO), vasoactive drugs, and other rescue treatments, the patient's condition did not improve. Ultimately, she gave up treatment and was automatically discharged on the eleventh day of admission. She eventually died on the seventh day after her discharge from the RICU.

**Patient 3** was a 54-year-old male farmer from Henan Province, China who was admitted to the nephrology department for “double lower extremity edema for 4 months.” The main clinical symptoms of the patient were fever, chest tightness, and dyspnea. The patient's body temperature peaked at 39°C. He had been diagnosed with Nephrotic Syndrome (NS) 2 months ago and had been using methylprednisolone for a long time. His case was additionally complicated by hypertension and type II diabetes. On the fourth day of admission, the patient's symptom of chest tightness worsened. Oxygen saturation decreased to 55% and the patient was urgently transferred to ICU.

The following abnormal laboratory test results were obtained: red blood cell count, 3.17 × 10^12^ cells/L; hemoglobin, 107.2 g/L; lactate dehydrogenase, 2,150 U/L; procalcitonin,.424 ng/ml; C-reactive protein, 26.81 mg/L; erythrocyte sedimentation rate, 132 mm/h; plasma β-(1, 3)-D glucan, 238 pg/ml; EB virus and cytomegalovirus IgG positive; EB virus and Herpes simplex II virus IgM weakly positive; and absolute count of CD4 cells, 212/ul.

**Figure 4:**
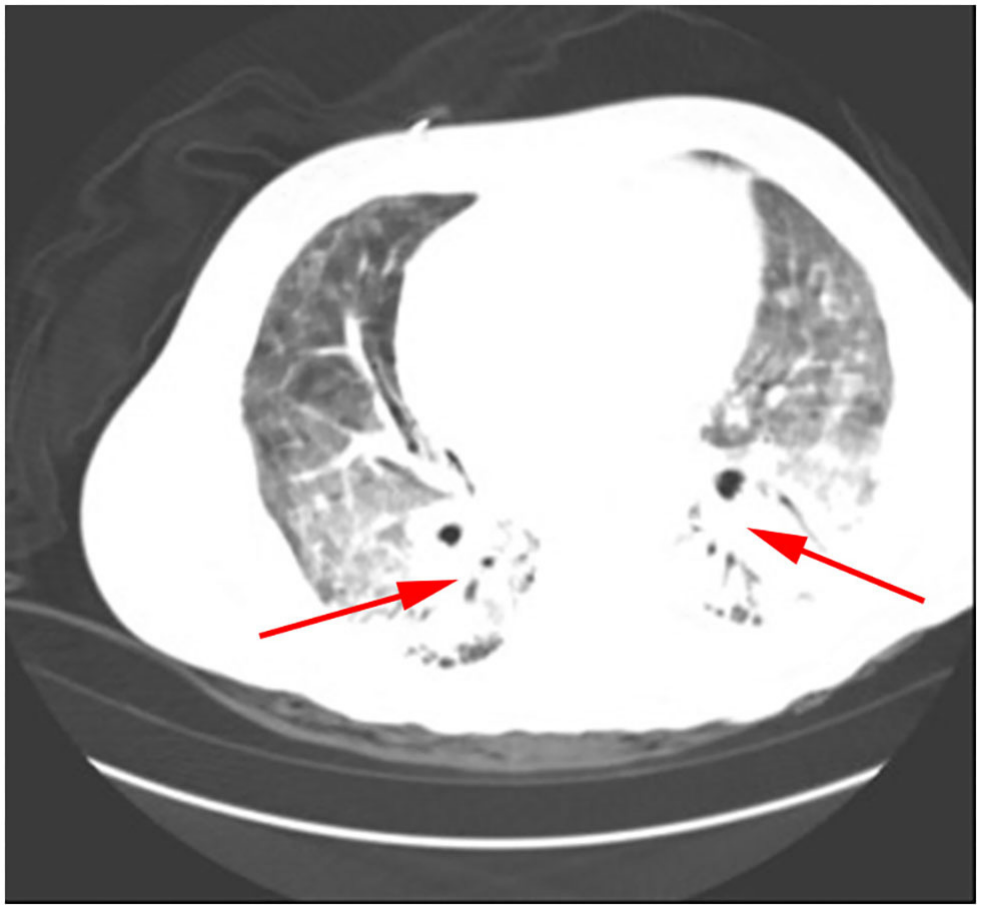
*P. jirovecii* appearances on Chest CT scan in patient 3 as a large number of meshwork shadows and multiple small nodules in both lungs.

*Klebsiella pneumoniae* were detected by traditional microbiological testing. Chest CT showed large meshes in both lungs with multiple nodules (Figure 4). BALF was collected for examination on the seventh day of admission. GMS (Figure 2C) and mNGS all detected *P. jirovecii*, but the detection period of mNGS was 1 day shorter than that of GMS. mNGS results showed that sequence number of *P. jirovecii* was 34 (Table 2). Based on the GMS and mNGS results, the patient was diagnosed with PJP. At the beginning of treatment, the patient was treated with tigecycline, biapenem, penciclovir, voriconazole, methylprednisolone, and other symptomatic treatments. When the results of mNGS were showed, specific anti *P. jirovecii* drugs were given. Unfortunately, the progress of this patient was the same as that of the previous patients. Even upon mechanical ventilation, continuous renal replacement therapy (CRRT), and other rescue treatments, there was still no improvement. Ultimately, he gave up treatment and was automatically discharged on the eleventh day of admission.

**Patient 4** was a 58-year-old male farmer from Henan Province, China who was admitted to respiratory department for “fever for 10 days, chest tightness 1 week.” The main clinical symptoms of the patient were chest tightness, dyspnea, and fever, with his body temperature peaking at 38.5°C. He had been diagnosed with NS for 3 months and had been using methylprednisolone and tacrolimus for a long undisclosed time. On the third day of admission, the patient's symptom of chest tightness worsened, oxygen saturation was measured at 78–80%, and the patient was urgently transferred to RICU.

The following abnormal laboratory test results were obtained: neutrophil percentage, 91.6%; lymphocyte percentage, 7.7%; lactate dehydrogenase, 2,150 U/L; plasma β-(1, 3)-D glucan, 244.5 pg/ml; procalcitonin,.59 ng/ml; C-reactive protein, 159 mg/L; erythrocyte sedimentation rate, 24 mm/h; EB virus IgG positive; Interleukin 6, 42.16 pg/ml; Interleukin 10, 8.08 pg/ml; percentage of total T lymphocytes, 68.12%; and absolute number of helper/inducible T lymphocytes, 51.4/μl.

*Candida glabra* was detected by traditional microbiological testing. Chest radiograph revealed multiple large flaps of exudate in both lungs. BALF was collected for examination on the third day after admission. GMS (Figure 2D) and mNGS all detected *P. jirovecii*, but the detection period of mNGS was, as before, 1 day shorter than that of GMS. mNGS results showed that sequence number of *P. jirovecii* was 279 (Table 2). Based on the GMS and mNGS results, the patient was diagnosed with PJP. SMZ-TMP was already empirically applied on the day of admission and the patient was also given imipenem, penciclovir, caspofungin, gamma globulin, methylprednisolone, and other symptomatic treatments. Regretfully, the progress of this patient was the same as that of all the other patients. There was still no improvement upon mechanical ventilation and other rescue treatments. He eventually gave up treatment and was automatically discharged.

## Discussion

With the aging of the population, increased occurrence of organ transplantation, and wide application of antibiotics, anti-tumor drugs, glucocorticoids (GC), and ISAs, the incidence and mortality of PJP in non-HIV-infected patients have been increasing every year (14, 15). In our study, all four patients were over 50 years old. Each had conditions that necessitated long-term GC or ISA therapy. Laboratory results showed a reduced lymphocyte percentage and a low CD4 + count. Studies have shown that patients with impaired immune function are prone to various infections and that the infection rate is as high as 25–50% (16). Pulmonary fungal infections are one of the most common types of infections in immunocompromised patients who require special attention (17–20). *P. jirovecii* is an opportunistic pathogen and is widely found in the lung tissue of mammals. When the body's immunity is low, it can lead to pathogenic fungal infections in the lungs. It is reported that the most important risk factors for developing PJP are the use of GCs, defective cell-mediated immunity, age, and comorbid pulmonary conditions (21–24). Consistent with previous literature reports, all patients in this study had the above risk factors for developing PJP.

Patients with HIV who are also infected with *P. jirovecii* usually present with fever, dry cough, dyspnea, and ground-glass opacity (GGO) imaging on chest CT. According to our observations, the main manifestations of the patients were fever, chest tightness, and dyspnea. In addition, chest CT showed multiple patchy, flocculent, and grid-like high-density shadows. Due to its atypical early symptoms and radiographic findings, it is easy for it to be clinically ignored, which eventually leads to delayed disease recognition and treatment. Although our clinicians did empirically administer antifungals, the outcome of patients was not satisfactory. All four of the patients in this study rapidly deteriorated and were transferred to the ICU for severe respiratory failure, pulmonary infection, and mechanical ventilation. Therefore, we need to identify the etiology as soon as possible so as to make an early diagnosis and begin early treatment.

*P. jirovecii* still cannot be cultured *in vitro*. Meanwhile, the gold standard for diagnosis of PJP is microscopic visualization of pneumocystis cysts or trophozoites in respiratory samples. In this study, we used traditional GMS as the diagnostic method, because GMS is the most common method for detecting pneumocystis cysts. The cyst wall may be stained dark gray or brown, which contrasts sharply against the background and makes it easy to be observed. However, the traditional GMS test cycle is long, and the detection rate is low (6.5–13.2%) (25, 26) and mainly depends on the expertise of the laboratory physician. In this study, we found two or more senior pathologists to observe *P. jirovecii* by GMS. Otherwise, we do not expect to have diagnosed PJP. Additionally, lactate dehydrogenase (LDH) is an important predictive marker for patients with PJP. LDH in patients with PJP was significantly higher than in non-PJP controls (27). Neutropenia is a risk factor of PJP. The 2019 American Society of Transplantation Infectious Diseases Community of Practice (AST IDCOP) guidelines suggested reinstituting PJP prophylaxis in patients with prolonged neutropenia (28). At the present time, the diagnosis of PJP remains challenging due to its non-specific clinical features, imaging manifestations, and the inadequate performance of conventional diagnostic methods. Therefore, the traditional methods cannot be applied to the clinical diagnosis of critically ill patients, and a new diagnostic method was urgently needed.

In our study, all four patients were in critical condition in the ICU, were immunosuppressed, and had underlying diseases. In addition, the traditional GMS method was immediately negative and had poor therapeutic effect, which indicated the need to use the mNGS method. mNGS is an unbiased approach that can theoretically detect all pathogens in a clinical sample and is especially suitable for rare, novel, and atypical etiologies of complicated infectious diseases (29). In this study, we used the mNGS detection method and found that the detection time of *P. jirovecii* by mNGS (24 h) was significantly faster than that of GMS (5–7 days). In addition, the results of mNGS were consistent with the traditional test results. Compared with the traditional GMS method, mNGS has absolute advantages. A retrospective study shows that mNGS reached a sensitivity of 100% in diagnosing PJP, which was remarkably higher than GMS staining (25.0%) and plasma β-(1,3)-D glucan (67.4%). The specificity of mNGS (96.3%) significantly surpassed plasma β-(1,3)-D glucan (81.4%) (30). So far, there have been 5 reports on successful use of mNGS to diagnose pulmonary infections involving *P. jirovecii* (31–35). A particular example would be from a study, where *P. jirovecii was* detected by mNGS in the patient from two different specimens. However, GMS only detected *P. jirovecii* in the second lung tissue sample submitted by this patient. This led to a longer time for a definitive diagnosis of PJP. Finally, the patient's condition worsened, and she died after the treatment failed. Although clinicians have given four patients anti-pneumocystis drug specific therapy, none of the four patients in this study had a good outcome. In this context, from our data analysis, we could see that the time from onset to diagnosis was more than 1 month. If the PJP mNGS detection method could be used to make a clear diagnosis when patients still have early-stage symptoms, then it will greatly reduce the mortality of this condition.

On the other hand, Karius test is a novel technique that uses open-ended next generation sequencing (NGS) of circulating microbial cell-free DNA (cfDNA). From a single blood sample, Karius test can non-invasively and rapidly detect over 1,000 pathogens that cause both deep-seated and bloodstream infections (31, 36). The test identified uncommon and difficult to diagnose organisms such as Pneumocystis jirovecii, Nocardia, Legionella, and Toxoplasma, resulting in rapid antimicrobial interventions. A recent study showed that the sensitivity and specificity of plasma cfDNA sequencing to detect *P. jirovecii* infection was 83.3 and 100%, respectively (37). Nevertheless, both of mNGS and Karius test still have some limitations. Firstly, the costs are relatively higher than GMS staining (600 vs. 100$), and it is not currently covered by medical insurance. Secondly, there is no standard interpretation of an mNGS or Karius test report and it is impossible to distinguish between colonized bacteria or infected bacteria. Thirdly, one disadvantage of Karius test was its false-positive rate. In patients with hematologic malignancies, chemotherapeutic treatment commonly causes mucosal barrier injury, leading to potential translocation of the microbial DNA across the mucosal membrane (38). Thus, we still cannot use mNGS as a complete substitute for traditional staining microscopy.

Overall, we can conclude that mNGS can be used to diagnose PJP, and that it is more rapid and efficient than traditional detection methods. Consideration of symptoms, laboratory testing, and imaging examination should be used in addition to mNGS test results to make comprehensive and accurate clinical judgments.

## Data Availability Statement

The original contributions presented in the study are included in the article/supplementary material, further inquiries can be directed to the corresponding author/s.

## Ethics Statement

The studies involving human participants were reviewed and approved by the Institutional Review Board of the First Affiliated Hospital of Zhengzhou University. The patients/participants provided their written informed consent to participate in this study.

## Author Contributions

MY, HN, and LX analyzed and interpreted patient data. XL and JZ wrote the manuscript. XL revised the article. WM and MY analyzed the genomics data. All authors have read and approved the final manuscript.

## Conflict of Interest

The authors declare that the research was conducted in the absence of any commercial or financial relationships that could be construed as a potential conflict of interest.

## Publisher's Note

All claims expressed in this article are solely those of the authors and do not necessarily represent those of their affiliated organizations, or those of the publisher, the editors and the reviewers. Any product that may be evaluated in this article, or claim that may be made by its manufacturer, is not guaranteed or endorsed by the publisher.
